# Production and Catalytic Properties of Amylases from* Lichtheimia ramosa* and* Thermoascus aurantiacus* by Solid-State Fermentation

**DOI:** 10.1155/2016/7323875

**Published:** 2016-06-20

**Authors:** Ana Paula Aguero de Oliveira, Maria Alice Silvestre, Nayara Fernanda Lisboa Garcia, Heloíza Ferreira Alves-Prado, André Rodrigues, Marcelo Fossa da Paz, Gustavo Graciano Fonseca, Rodrigo Simões Ribeiro Leite

**Affiliations:** ^1^Laboratory of Enzymology and Fermentation Processes, Faculty of Biological and Environmental Sciences, Federal University of Grande Dourados (FCBA/UFGD), Rodovia Dourados/Itahum, km 12, 79804-970 Dourados, MS, Brazil; ^2^Faculty of Engineering, Department of Phytotechnology, Food Technology and Social Economy, São Paulo State University (FEIS/UNESP), Avenida Brasil, No. 56, 15385-000 Ilha Solteira, SP, Brazil; ^3^Laboratory of Fungal Ecology and Systematics, Biosciences Institute, Department of Biochemistry and Microbiology, São Paulo State University (IB/UNESP), Avenida 24A, No. 1515, 13506-900 Rio Claro, SP, Brazil; ^4^Laboratory of Bioengineering, Faculty of Biological and Environmental Sciences, Federal University of Grande Dourados (FCBA/UFGD), Rodovia Dourados/Itahum, km 12, 79804-970 Dourados, MS, Brazil

## Abstract

The present study compared the production and the catalytic properties of amylolytic enzymes obtained from the fungi* Lichtheimia ramosa* (mesophilic) and* Thermoascus aurantiacus* (thermophilic). The highest amylase production in both fungi was observed in wheat bran supplemented with nutrient solution (pH 4.0) after 96 hours of cultivation, reaching 417.2 U/g of dry substrate (or 41.72 U/mL) and 144.5 U/g of dry substrate (or 14.45 U/mL) for* L. ramosa* and* T. aurantiacus*, respectively. The enzymes showed higher catalytic activity at pH 6.0 at 60°C. The amylases produced by* L. ramosa* and* T. aurantiacus* were stable between pH 3.5–10.5 and pH 4.5–9.5, respectively. The amylase of* L. ramosa* was stable at 55°C after 1 hour of incubation, whereas that of* T. aurantiacus* maintained 60% of its original activity under the same conditions. Both enzymes were active in the presence of ethanol. The enzymes hydrolyzed starch from different sources, with the best results obtained with corn starch. The enzymatic complex produced by* L. ramosa* showed dextrinizing and saccharifying potential. The enzymatic extract produced by the fungus* T. aurantiacus* presented only saccharifying potential, releasing glucose monomers as the main hydrolysis product.

## 1. Introduction

Population growth prompts the discovery of new food and energy sources, which will be possible with the best use of the polysaccharides that constitute the vegetal biomass [1, 2]. Starch is one of the major vegetable reserve compounds, composed of glucose units linked by glycosidic bonds. This polysaccharide has been used as a major energy source in several trophic levels of the food chain [3].

The saccharification of starch enables the obtainment of maltose or glucose syrups as sweeteners for the food industry and for the production of ethanol derived from fermentation processes [1, 4]. Hydrolysis of starch by enzymatic methods has advantages compared to that with chemical methods, such as operating under mild conditions of pH and temperature, preventing equipment corrosion, and subsequent neutralization steps. Enzymes have substrate specificity, eliminating the formation of undesirable by-products, commonly observed in acid hydrolysis [5, 6].

In addition to the production of biofuels from starch sources, amylases are applied in bread-making, in detergents for industrial cleaning, and in the degumming processes of textile fibers. Thus, amylases represent about 25–33% of the global enzyme market [7, 8]. However, the production cost of enzymes on an industrial scale is still elevated. It is estimated that the formulation of microbial culture medium represents about 30–40% of the final cost of an enzyme [9].

The need for reducing the production costs of industrial enzymes encourages the search for low-cost microbial culture medium. In this regard, agroindustrial residues have been used as substrates for microbial-derived enzymes under solid-state fermentation (SSF) [10, 11] and several published works have used this process for the production of various enzymes [6, 12–14].

Solid-state fermentation has some similarities to the natural environment of the microorganisms, especially for the filamentous fungi, as a promising alternative for the cultivation of these organisms. However, some disadvantages can also be highlighted owing to the low accessibility of the substrates and low homogeneity of the medium, making it difficult to control the operating parameters (pH, temperature, moisture, and others). These aspects have stimulated research to improve the use of SSF for industrial processes [10, 11].

In this study, we compared the production and the catalytic properties of amylases produced by the filamentous fungi* Lichtheimia ramosa* and* Thermoascus aurantiacus*, cultivated by SSF in agroindustrial residues. These strains were isolated in the Midwest Brazilian region (less explored for microbial bioprospecting) and selected for amylase production.

## 2. Materials and Methods

### 2.1. Microorganisms

The filamentous fungi* L. ramosa* (mesophilic species) and* T. aurantiacus* (thermophilic species) were assessed for amylase production. The microorganism* L. ramosa* was isolated from sugarcane bagasse processed in a sugarcane ethanol plant [15]. The fungus* T. aurantiacus* was isolated from leaf litter of Atlantic seasonal forest fragment located in Dourados, Mato Grosso do Sul State, Brazil. Working-stock cultures of both fungi were incubated on Sabouraud dextrose agar medium at 4°C.

### 2.2. Inoculum

The fungi were cultivated in 250 mL Erlenmeyer flask containing 40 mL of Sabouraud dextrose agar, incubated for 48 hours at 28°C and 45°C for* L. ramosa* and* T. aurantiacus*, respectively. The fungal suspensions were obtained by gently scraping the surface of the culture medium and putting it into 30 mL of nutrient solution (0.1% ammonium sulfate, 0.1% magnesium sulfate heptahydrate, and 0.1% ammonium nitrite, Merheb-Dini et al. [16]). The fungi were inoculated in the agroindustrial residues by transfer of 5 mL of the microbial suspension.

### 2.3. Solid-State Fermentation

The fungi were cultivated in 250 mL Erlenmeyer flasks containing 5 g of different agroindustrial residues (wheat bran, soy bran, corn cob, corn straw, rice peel, and sugarcane bagasse). Other fermentative parameters were also varied in this study such as the nutrient solution pH (3.0–5.0), the initial moisture (50–90%), and the cultivation time (24–168 h). The best growth conditions established in each step were adopted in subsequent assays. All material was autoclaved for 20 min at 121°C. All assays were performed in duplicate and the values described represent the respective averages.

### 2.4. Enzyme Extraction

Enzymes were obtained from the fermented residues by adding 50 mL of distilled water and shaking constantly at 100 rpm for 1 h. The samples were filtered through nylon cloth and centrifuged at 3,000 ×g for 5 min at 5°C. The supernatant was considered the extracellular enzymatic extract and used in the subsequent steps.

### 2.5. Determination of Amylase Activity

The enzyme activity was determined by adding 0.1 mL of enzymatic extract to 0.9 mL of sodium acetate buffer (0.1 M, pH 5.0) containing 1% corn starch. After 10 minutes of reaction at 50°C, the reducing sugars released were quantified by measuring the absorbance at 540 nm by the DNS method (3,5-dinitrosalicylic acid) [17]. One unit of enzymatic activity was defined as the amount of enzyme required to release 1 *µ*mol of the product per minute of reaction.

### 2.6. Biochemical Characterization of the Amylases Produced by SSF

#### 2.6.1. Effect of pH and Temperature

The optimum pH was determined by measuring the enzymatic activity at 50°C with different pH conditions (3.0–8.0) by using McIlvaine buffer 0.1 M. The optimum temperature was determined by measuring the enzymatic activity from 30 to 75°C at the respective optimal pH of each enzyme. The enzymatic stability to variations in pH was assessed by incubating the enzymes at 25°C for 24 h at different pH range. The following buffers were used: McIlvaine 0.1 M (3.0–8.0), Tris-HCl 0.1 M (8.0–8.5), and Glycine-NaOH 0.1 M (8.5–10.5). The thermostability of the enzymes was assessed by incubating for 1 h at different temperatures (30–75°C). The residual activity was determined under optimum conditions of pH and temperature [18].

#### 2.6.2. Effect of Ethanol on Enzymatic Activity

Enzyme activity was quantified by adding different concentrations of ethanol (0–30%) to the reaction mixture. The assays were performed at 50°C in McIlvaine buffer (0.1 M, pH 6.0) containing 1% corn starch [18].

#### 2.6.3. Enzymatic Hydrolysis of Starch Derived from Several Vegetal Sources

Enzymatic assays were performed using potato, corn, wheat, and cassava starch (1%) as substrates. The enzymatic reactions were performed in McIlvaine buffer 0.1 M (pH 6.0). The sugar released was quantified using the DNS method [17].

#### 2.6.4. Dextrinization Potential of Enzymatic Extracts

Dextrinizing activity was assessed using corn starch (1%) as enzymatic substrate in McIlvaine buffer 0.1 M (pH 6.0) and the iodometric methods described by Fuwa [19] and Pongsawadi and Yagisawa [20]. The reaction mix contained 0.1 mL of enzymatic extract in 0.3 mL of buffer solution containing starch. After 10 minutes at 60°C, the reaction was stopped by adding 4 mL of 0.2 M HCl. Finally, 0.5 mL of reactive iodine and 10 mL of distilled water were added. The absorbance was quantified at 700 nm. One unit of activity was defined as the amount of enzyme required to reduce the intensity of the blue color of the starch iodine complex by 10% per minute of reaction.

#### 2.6.5. Saccharification Potential of Enzymatic Extracts

The saccharifying activity of the enzymatic extracts was assessed by the glucose oxidase/peroxidase method, using 1% corn starch as enzymatic substrate in McIlvaine buffer 0.1 M (pH 6.0) [21]. The reaction mixture contained 0.1 mL of the enzymatic extract in 0.4 mL of buffer solution containing starch. After 10 minutes at 60°C, the reaction was stopped in ice bath. The glucose released was quantified using an enzymatic colorimetric kit (*Glicose-PP*,* Gold Analisa Diagnóstica Ltda*,* Brazil*). The absorbance was measured at 505 nm. One unit of enzyme activity was defined as the amount of enzyme required to release 1 *µ*mol of glucose per minute of reaction.

### 2.7. Statistical Analysis

All experiments were performed as duplicates and the results are presented as the mean of two independent tests. Statistical analysis of the data included a one-way ANOVA followed by Tukey's test with a 5% significance level.

## 3. Results and Discussion

### 3.1. Amylase Production by SSF

Among the tested substrates, both microorganisms showed higher production of amylase, on wheat bran, reaching 320.4 U/g of dry substrate (or 32.04 U/mL) for* L. ramosa* and 44.2 U/g of dry substrate (or 4.42 U/mL) for* T. aurantiacus* (Table 1). Regarding the analysis of variance, the results were significant with ANOVA *P* value being < 0.0001, considered extremely significant.

In general, wheat bran has the highest amount of macro- and micronutrients when compared to other agricultural residues such as sugarcane bagasse, rice straw, wheat straw, and rice bran [22]. According to Haque et al. [23], wheat bran consists of a complex substrate rich in proteins, carbohydrates, minerals, lipids, and vitamins favoring microbial growth and enzyme production. Additionally, Kunamneni et al. [24] reported that wheat bran was the best substrate for amylase production by the fungus* Thermomyces lanuginosus*. Moreira et al. [25] demonstrated a higher amylase production when different* Aspergillus* species were cultivated on wheat bran, increasing the production of the enzyme by up to 10-fold compared to that obtained by growth on other residues.

**Figure 1:**
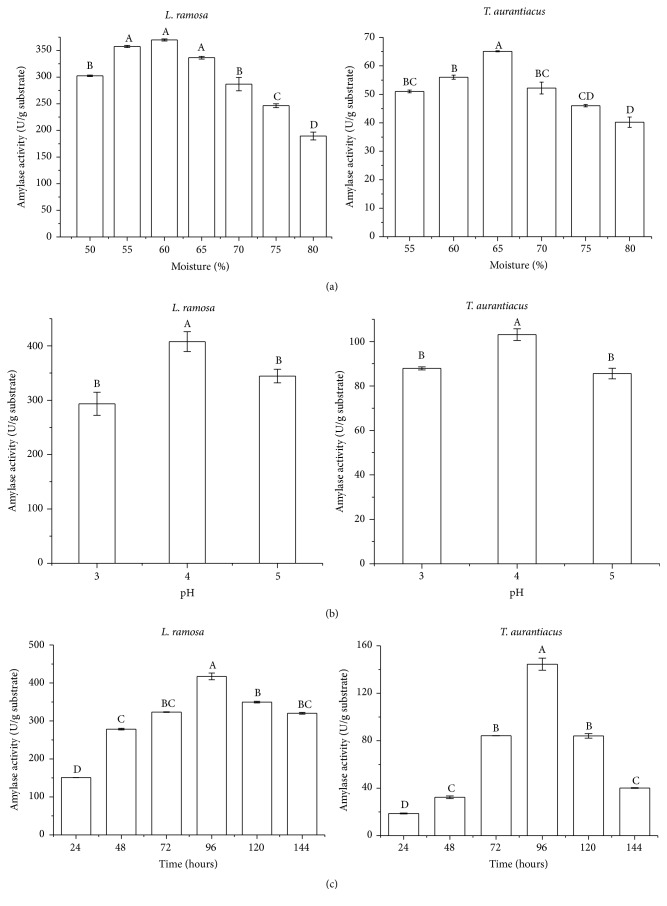
Amylase production by* L. ramosa* and* T. aurantiacus* by solid-state fermentation in wheat bran. (a) Influence of initial substrate moisture; (b) influence of initial cultivation pH; (c) influence of cultivation time. Average production with different letters indicates significant differences according to Tukey test.

Considering the data presented in Table 1, wheat bran was used in subsequent cultivation steps to evaluate different fermentative parameters for amylase production, such as initial moisture, pH of the nutrient solution, and cultivation time. There are no significant differences between 55 and 65 but it is clear that highest amylase production was obtained when* L. ramosa* was cultivated at 60% moisture (w/v), with a maximum value of 369.8 U/g of dry substrate (or 36.98 U/mL). The fungus* T. aurantiacus* increased amylase production, about 65.1 U/g of dry substrate (or 6.51 U/mL), when grown on wheat bran containing 65% moisture (w/v) (Figure 1(a)). *P* value is 0.0004, considered extremely significant.

Moisture in the substrate is a fundamental parameter in SSF. The medium should contain enough moisture to allow microbial physiological activities but it cannot exceed the substrate absorption limit, leaving free water among the solid particles. Excess moisture results in the decrease of porosity, affects gas exchange, and favors bacterial contamination, disfavoring the growth and enzyme production [26, 27].

Significant difference was found in the study of nutrient solution pH (3.0–5.0). The highest amylase production was obtained in cultures supplemented with nutrient solution adjusted to pH 4.0 (Figure 1(b)), with enzymatic activity of 407.9 U/g of dry substrate (or 40.79 U/mL) for* L. ramosa* and 103.1 U/g of dry substrate (or 10.31 U/mL) for* T. aurantiacus*. Omemu et al. [28] reported that pH 4.0 was optimal for amylase production by* Aspergillus niger* and the same was observed by Gomes et al. [29] for* Aspergillus flavus*.

The last fermentative parameter evaluated in this study was the cultivation time. All parameters defined as optimum in the previous assays were employed for this experiment. Both organisms had statistical significance for the highest enzymatic production at 96 hours of cultivation, at which point 417.2 U/g of dry substrate (or 41.72 U/mL) for* L. ramosa* and 144.5 U/g of dry substrate (or 14.45 U/mL) for* T. aurantiacus* were obtained (Figure 1(c)).

The results showed a considerable decrease in enzymatic activity, after reaching the production peak (Figure 1(c)). This reduction is likely due to the prolonged incubation period, which may have led to (i) nutrient depletion in the culture medium, (ii) variations in the pH due to the microbial metabolic activity, or (iii) the presence of proteolytic enzymes [30, 31].

After the optimization of the fermentative process, amylase production by* L. ramosa* rose from 320.4 to 417.2 U/g of dry substrate, an increase of approximately 30% (Table 1 and Figure 1(c)). On the other hand, the most notable result was the increase of amylase production of* T. aurantiacus* from 44.2 to 144.5 U/g of dry substrate, representing a gain of more than 200% when compared to the initial cultures (Table 1 and Figure 1(c)).

Considering the several studies on amylase production, our results are promising. Bhatti et al. [32] reported the glucoamylase production (about 61.35 U/g of dry substrate) when cultivated in* Fusarium solani* by SSF using wheat bran as substrate. Moreira et al. [25] obtained amylases by SSF using wheat bran as substrate with no additional carbon sources, achieving approximately 350, 240, and 210 U/g of dry substrate by the fungi* Aspergillus flavus*,* Aspergillus fumigatus*, and* Aspergillus tamari*, respectively. The authors reported a 1.5- to 2-fold increase in the production of amylases in wheat bran supplemented with various carbon sources. Kunamneni et al. [24] obtained maximum amylase production (about 534 U/g of substrate) after 120 hours in SSF for* Thermomyces lanuginosus*, in wheat bran supplemented with 1% soluble starch.

### 3.2. Biochemical Characterization of the Amylases Produced by SSF

#### 3.2.1. Effect of pH and Temperature

**Figure 2:**
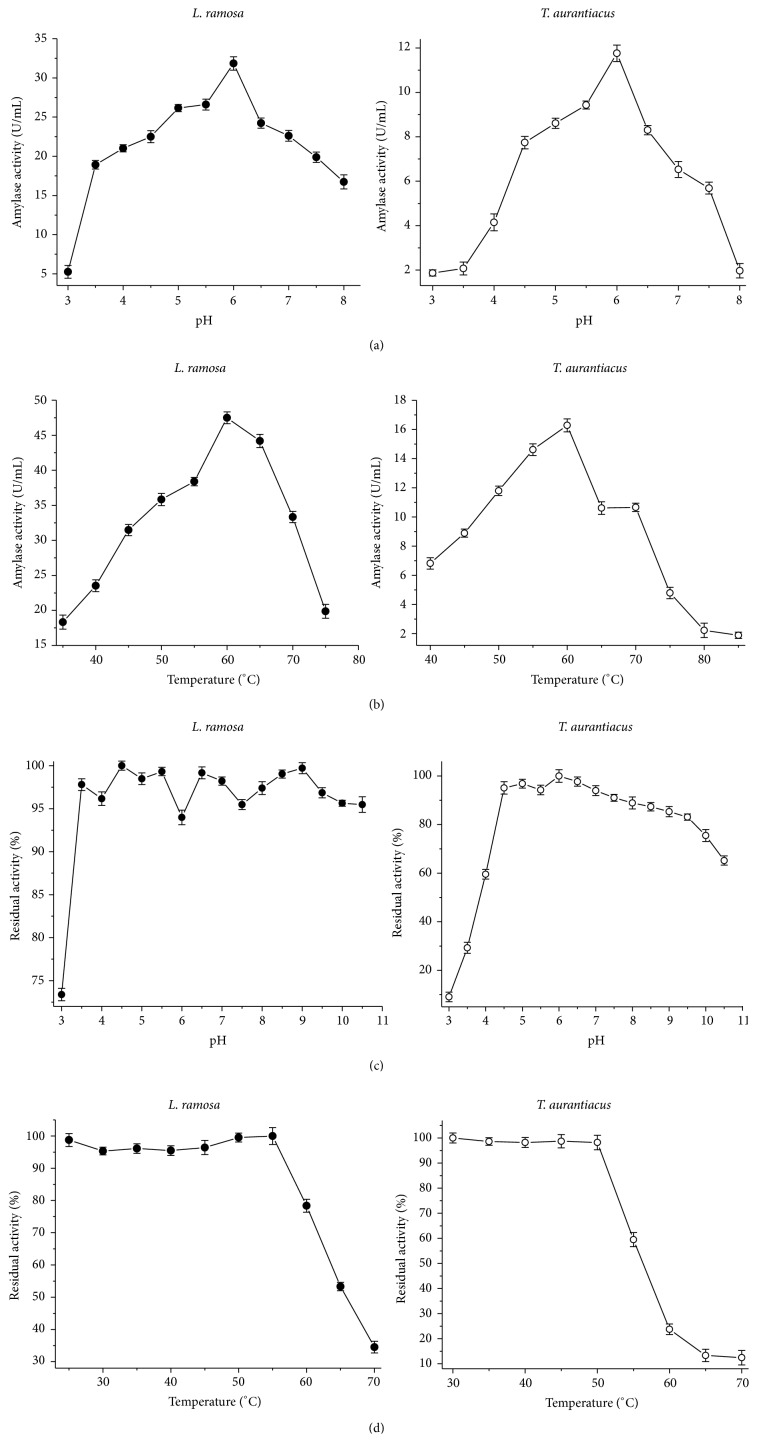
Effect of pH and temperature on amylase activity of* L. ramosa* and* T. aurantiacus*. (a) Amylase activity at different pHs and (b) at different temperatures; (c) amylase residual activity after 24 h at different pHs and (d) after one hour at different temperatures (each data point was the average of two replicate determinations, and the error bars show the data ranges).

The amylases produced by both fungi showed optimum activity at pH 6.0 and at 60°C (Figures 2(a) and 2(b)).

The enzymes evaluated in this study showed maximum activity at temperatures higher than those described by Alva et al. [30] for amylases produced by* Aspergillus* in SSF. This suggests high structural stability of enzymes produced by* L. ramosa* and* T. aurantiacus*. However, our results showed similarity to previously published articles. Giannesi et al. [33] reported that amylase obtained from different microbial sources may have optimum pH between 4.5 and 7.0. The same authors described 60°C as the optimum temperature for *α*-glucosidase purified from enzymatic extracts of* Chaetomium thermophilum* var.* coprophilum*.

Both enzymes were stable in a wide pH range. The amylase produced by* L. ramosa* maintained its activity at pH from 3.5 to 10.5 (Figure 2(c)) and the enzyme produced by* T. aurantiacus* was stable at pH 4.5 to 9.5 (Figure 2(c)). According to Michelin et al. [34] the maintenance of enzymatic activity in a wide pH range is an advantage for application in industrial processes, because it requires lower pH adjustments between the sequential treatments of liquefaction and saccharification of starch.

The amylase produced by* L. ramosa* retained its catalytic activity after 1 hour at 55°C and 75% of its original activity when incubated for the same period at 60°C (Figure 2(d)). The amylase produced by* T. aurantiacus* remained stable after 1 hour at 50°C; when the temperature was raised to 60°C, the enzyme showed only 25% of its initial activity (Figure 2(d)).

The results presented in Figure 2 indicate that the amylase produced by the mesophilic fungus* L. ramosa* had a higher structural stability compared to the enzyme produced by the thermophilic fungus* T. aurantiacus*. This characteristic is very appreciable in industrial applications, considering that industrial environment differs significantly from laboratory conditions, in regard to the control of pH and temperature. The structural stability of the enzymes is indispensable to withstand variations in these parameters during different processes. According to Bruins et al. [35], there is no complex structural system that distinguishes a stable protein from another with less stability. Small molecular alterations as the number of hydrogen and disulfide bonds, folding, and hydrophobicity degree of the molecule and the amount of ionic linkages can produce large modifications in the stability of a protein.

Although it is not usual to observe high thermostability in enzymes produced by mesophilic microorganisms, results from previous studies support this possibility [18]. Gomes et al. [29] reported thermostability ranging from 10 to 60°C for amylases produced by* Aspergillus flavus* (mesophilic) and from 10 to 40°C for amylases of* Thermomyces lanuginosus* (thermophilic).

#### 3.2.2. Effect of Ethanol on Enzymatic Activity

**Figure 3:**
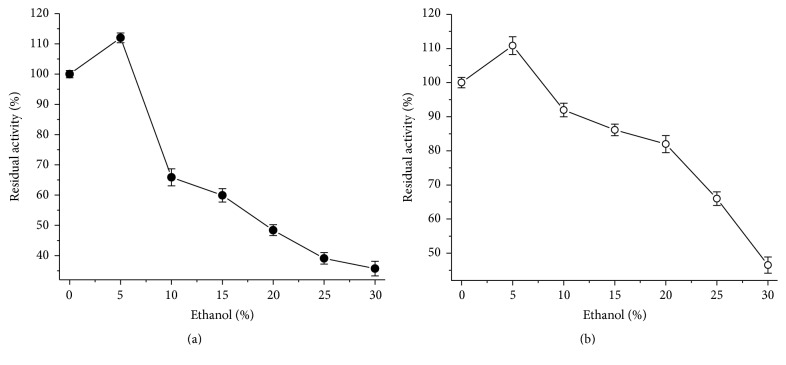
Effect of ethanol on amylase activity. (a)* L. ramosa*; (b)* T. aurantiacus* (each data point was the average of two replicate determinations, and the error bars show the data ranges).

Ethanol inhibition is a strong trend in the study of certain enzymes, since they can be exposed to substantial concentrations of alcohol for various industrial applications [6]. The results demonstrate that amylase produced by* L. ramosa* showed residual activity around 65% and* T. aurantiacus* amylase showed greater than 90% residual activity when incubated at concentrations of 10% ethanol (Figure 3).

This data reveals that both enzymes have potential for use in alcoholic fermentation processes derived from starch sources. In conventional alcoholic production processes, concentrations higher than 10% ethanol are extremely harmful to the fermenters organisms [36]. The increase of catalytic potential by ethanol may be associated with transferase activity, with ethanol being used as an intermediate acceptor by the enzyme; thus, resulting in increased reaction rate [18, 37].

#### 3.2.3. Enzymatic Hydrolysis of Starch Derived from Several Vegetal Sources

**Figure 4:**
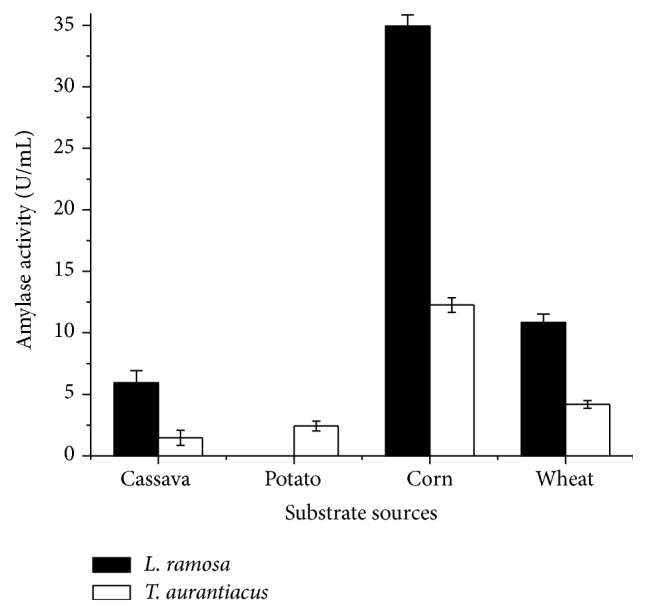
Enzymatic hydrolysis of starch derived from several vegetal sources (DNS method) (each data point was the average of two replicate determinations, and the error bars show the data ranges).

The action of amylolytic enzymes on starch from different vegetal sources was evaluated. Enzymatic extracts showed the highest catalytic potential on corn starch, obtaining 34.94 U/mL and 12.26 U/mL for amylases produced by* L. ramosa* and* T. aurantiacus*, respectively (Figure 4).

Differences in the action of amylolytic enzymes may be related to the composition of the starch molecules, in particular, the amylose content and the extent of their chains. The lipid, protein, and mineral levels may also influence the enzyme activity. Another factor that may be related to the susceptibility of starch granules to enzymatic attack is the pore-size present on their surface [38].

The content of amylose and amylopectin varies according to the botanical source, providing specific characteristics to starch, reflecting in the granule architecture and its textural properties [39]. The corn starch has a higher amount of amylose, compared to other starches and consequently lower amylopectin content, favoring enzymatic degradation. The amylopectin exhibits highly ramified structure with high molecular weight, disfavoring the catalytic performance of amylolytic enzymes [40].

#### 3.2.4. Dextrinization and Saccharification Potential of Enzymatic Extracts

**Figure 5:**
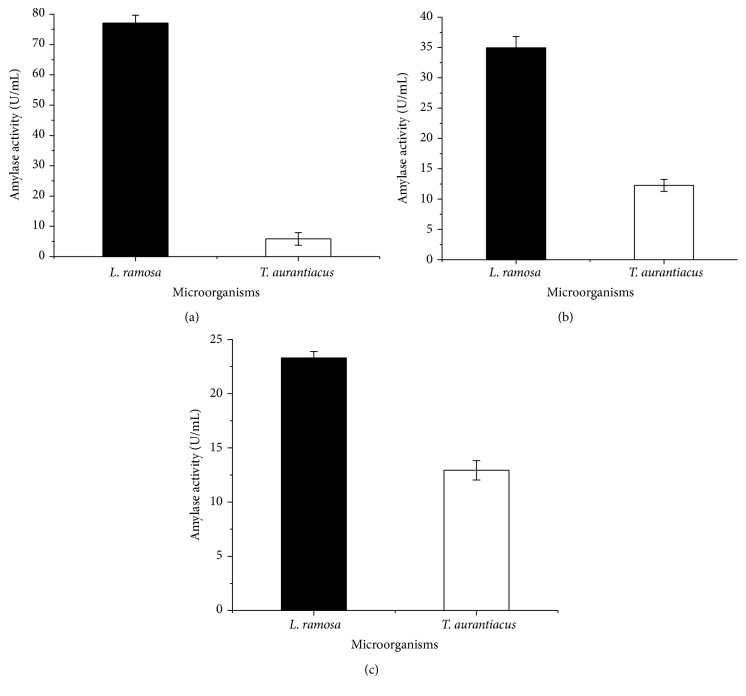
Enzymatic modifications of the corn starch molecule. (a) Quantification of dextrinizing activity using the iodometric method (reduction in the starch polymerization degree); (b) quantification of sugars and reducing ends using the DNS method; (c) quantification of glucose using the glucose/oxidase method (each data point was the average of two replicate determinations, and the error bars show the data ranges).

Comparing the action of enzymatic extracts on the starch molecule by different colorimetric methods, we observed that the enzymatic extract produced by* L. ramosa* showed high depolymerizing potential (dextrinizing activity), resulting in high amount of reducing chain ends (Figures 5(a) and 5(b)), some of which being glucose monomers, measured by glucose oxidase method, specific for determining glucose (Figure 5(c)).

These results suggest that the enzymatic extract obtained by* L. ramosa* under the conditions described above shows synergic activity of dextrinizing and saccharifying enzymes (endo and exoamylases production). The synergic action of amylases produced by a single microorganism is not commonly found. However, previous studies confirm this possibility. Silva et al. [41] reported the production of dextrinizing and saccharifying enzymes by the filamentous fungus* Rhizomucor pusillus*. Ezeji and Bahl [42] also reported the potential for producing *α*-amylase and glucoamylase by the bacterium* Geobacillus thermodenitrificans*.

The enzymatic extract produced by* T. aurantiacus* presented low depolymerizing potential (Figure 5(a)). Similar amounts of total reducing sugar (quantified by DNS) and free glucose (glucose oxidase quantified) suggest a predominantly exoamylolytic activity (Figures 5(b) and 5(c)) with glucose monomers as the main product, a typical catalytic property of glucoamylase (EC 3.2.1.3) or *α*-glucosidase (EC 3.2.1.20). Previous studies confirm these results. Carvalho et al. [43] reported *α*-glucosidase production by submerged fermentation of the fungus* T. aurantiacus*.

## 4. Conclusions

The two examined strains showed potential for amylase production by SSF, using wheat bran as substrate. However, the production of amylase by* L. ramosa* was considerably higher, compared to that of* T. aurantiacus*. The enzyme produced by* L. ramosa* also showed greater stability at different pHs and temperatures, characteristics that are very appreciable for industrial application. Enzymes of both microorganisms retained their catalytic activities in alcoholic conditions, so they can be applied to processes for obtaining ethanol from starch sources. Another interesting characteristic observed in the enzymatic extract produced by* L. ramosa* is the synergic potential of liquefaction and saccharification of starch, indicating the presence of endo and exoamylases in its composition. The properties described for amylase obtained from the fungus* L. ramosa* highlight the importance of this work, considering that this fungal species is still less explored for amylase production.

## Figures and Tables

**Table 1:** Amylase production by solid-state fermentation in several agroindustrial residues after 120 hours of cultivation with 75% moisture at pH 5.0. Average production with different letters indicates significant differences (*P* < 0.0001) according to Tukey test.

Substrate	*Lichtheimia ramosa* (U/g dry substrate)	*Thermoascus aurantiacus* (U/g dry substrate)
Sugarcane bagasse	—	3.7 ± 0.0^c^
Corn straw	8.8 ± 0.5^b^	6.2 ± 0.0^bc^
Soy bran	10.5 ± 0.3^b^	7.6 ± 0.1^b^
Rice peel	3.9 ± 0.1^b^	4.7 ± 0.1^bc^
Corn cob	9.2 ± 0.5^b^	5.2 ± 0.9^bc^
Wheat bran	320.7 ± 6.1^a^	44.2 ± 1.0^a^
